# Molecular Dynamics Investigation of Giant Clustering in Small-Molecule Solutions: The Case of Aqueous PEHA

**DOI:** 10.1021/acs.jpcb.2c04489

**Published:** 2022-10-25

**Authors:** Nasser D. Afify, Carlos A. Ferreiro-Rangel, Martin B. Sweatman

**Affiliations:** School of Engineering, The University of Edinburgh, The King’s Buildings, Sanderson Building, Mayfield Road, Edinburgh EH9 3JL, United Kingdom

## Abstract

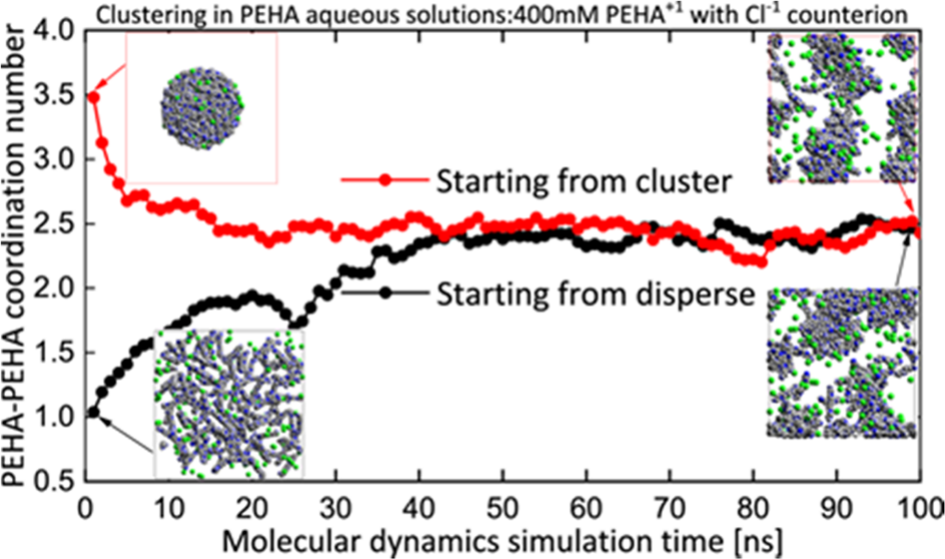

The importance of the formation of giant clusters in solution, in nature and industry, is increasingly recognized. However, relatively little attention has been paid to the formation of giant clusters in solutions of small, relatively soluble but nonamphiphilic molecules. In this work, we present a general methodology based on molecular dynamics that can be used to investigate such systems. As a case study, we focus on the formation of apparently stable clusters of pentaethylenehexamine (PEHA) in water. These clusters have been used as templates for the construction of bioinspired silica nanoparticles. To better understand clustering in this system, we study the effect of PEHA protonation state (neutral, +1, and +2) and counterion type (chloride or acetate) on PEHA clustering in dilute aqueous solutions (200 and 400 mM) using large-scale classical molecular dynamics. We find that large stable clusters are formed by singly charged PEHA with chloride or acetate as the counterion, although it is not clear for the case with acetate whether bulk phase separation, that might lead to precipitation, would eventually occur. Large clusters also appear to be stable for doubly charged PEHA with acetate, the less soluble counterion. We attribute this behavior to a form of complex coacervation, observed here for relatively small and highly soluble molecules (PEHA + counterion) rather than the large polyions usually found to form such coacervates. We discuss whether this behavior might also be described by an effective SALR (short-range attraction, long-range repulsion) interaction. This work might help future studies of additives for the design of novel bioinspired templated nanomaterials and of giant clustering in small-molecule solutions more generally.

## 1 Introduction

Giant equilibrium clusters occur in many large-molecule solutions.^1^ Well-known examples involve large biomolecules, including proteins (Meilhac and Destainville; Cardinaux et al.) and peptides^2−4^ (Viet et al.). The formation of giant equilibrium clusters in these systems is often explained in terms of the SALR (short-range attraction, long-range repulsion) model fluid, which is known to form a cluster fluid phase at low concentration.^5^ These giant SALR clusters are size-limited because growth of a bulk phase is arrested by the accumulation of repulsive (typically screened coulomb) interactions as cluster size increases. Giant equilibrium clusters are also known to occur for many combinations of oppositely charged polyelectrolytes.^6^ Here, the giant clusters, which typically appear as droplets, are often described as a microphase dispersion or complex coacervate. These fluid mixtures might also be described in terms of an effective one-component SALR model.^5^ Of course, large clusters are also known to occur in aqueous surfactant systems that can form micelles. In this case, cluster formation is driven instead by the amphiphilic nature of the solute and cluster size is limited by the geometry of the surfactant molecule.

However, since length scale in the SALR model is arbitrary, giant clusters might also occur for solutions of many small nonamphiphilic molecules. For such cases, the clusters might appear as nanoscale droplets and therefore be too small even for optical microscopy to image. This suggests it is possible that giant equilibrium clusters exist unnoticed in many small-molecule solutions. However, even when such nanodroplets are observed, via light-scattering experiments for example,^7^ they are sometimes dismissed as being the result of “impurities” rather than aggregates of the main solute. Given the importance of many small-molecule solutions, with applications across science and engineering, it is therefore useful to develop simulation methods able to investigate such cases.

It is important to distinguish between the giant clusters, or nanodroplets, in which we are interested with the much smaller-scale clustering observed in some other liquid mixtures, such as ethanol–water mixtures at high ethanol concentrations.^8^ The giant clusters of interest to us are typically nanometer- to micron-sized and freely dispersed within a solvent with a much lower concentration of solute. The droplets are typically well-defined, mobile, approximately spherical and relatively long-lasting liquid-like inclusions within a background phase. They have a clear maximum in their size distribution well above the solute size, which is separated from clusters of just a few solutes by a deep minimum. This indicates a high nucleation barrier likely exists for their formation. This situation is quite different to nanostructured liquids like ethanol–water, which display two interpenetrating percolating networks rich in either the solute or solvent with length scale ∼1 nm.

Aqueous glycine is one such case of a nonamphiphilic, small-molecule solution that exhibits giant equilibrium clusters.^7^ Being the simplest amino acid, glycine is important in biology and is considered a good model for studies of crystallization from solution. Light-scattering studies reveal an apparently equilibrium phase of giant clusters, or nanodroplets, at concentrations far below the solubility limit of the solid phase. Earlier work used molecular dynamics (MD) to investigate the possibility these nanodroplets were composed primarily of glycine.^9^ While this possibility could not be ruled out, it was suggested a more likely possibility is that they instead are formed mainly from a reaction product of glycine in aqueous solution, possibly diketopiperazine.

In this present work, we investigate another apparent case of giant clustering in an aqueous solution of small nonamphiphilic molecules, namely, pentaethylenehexamine (PEHA). Just as for the glycine case, we aim to discover whether the nanodroplets observed in experiments on aqueous PEHA consist primarily of the main solute, PEHA, or an impurity since this has important implications for how PEHA is used in biosilica nanoparticle production and for how biosilica nanoparticle properties can be controlled. In particular, we further develop the molecular dynamics methodology developed previously for aqueous glycine^9^ to study aqueous PEHA. Thus, together with our earlier work on aqueous glycine, the present study demonstrates an important tool and methodology for the investigation of the general phenomena of giant clustering in small-molecule solutions.

### 1.1 Aqueous PEHA

Polyamines are organic molecules with two or more amine groups that tend to protonate in aqueous solutions. They can form linear or branched polycations, are observed widely in nature, and are used extensively in industry.^10^ In fact, they are present in all tissues and cell types examined in animals and plants, although some bacteria and eukaryotic parasites do not synthesize them.^11,12^ The presence of polyamines is essential for cell growth.^13^

Pentaethylenehexamine (PEHA) is a relatively small and soluble polyamine with several important applications in a number of industries. For example, it is used as a hardener with epoxy resins in several industrial and consumer applications and it is an intermediate in the synthesis of several substances, such as chemicals that are mixed with asphalt to pave roads.^14^ PEHA also is used widely in the manufacture of lubricating oils and fuel additives and has applications in agricultural chemicals, fungicides, bactericides, wood preservatives, chelating agents, surfactants, mineral processing aids and polymers. However, our interest in PEHA here is due to its use as an additive in the synthesis of a novel nanomaterial, bioinspired nanosilica.^15,16^

The nanosilica particles studied by Patwardhan and others^17^ have “bioinspired” and “green” labels because their synthesis is loosely based on the formation process of silica-rich diatom shells (diatoms are one of the most numerous kinds of plankton, responsible for producing much of the world’s atmospheric oxygen). Moreover, synthesis is carried out rapidly in aqueous solutions at room temperature and near-neutral pH, in contrast to current commercial and most lab-based syntheses of porous silica which use high temperatures and high pH resulting in energy-intensive processes that create significant waste. The resulting nanomaterials have a wide range of potential applications, from drug delivery to water treatment.^17^

However, there remains some uncertainty about the role of small polyamines in the synthesis of these bioinspired nanosilicas. It is suggested that PEHA and its analogues form giant, stable, liquid-like clusters (or nanodroplets) in solution around which the silica nanoparticles grow. Essentially, the nanodroplets are thought to act as both template and catalyst in this process and are therefore considered essential for nanoparticle formation.^18,19^ The resulting solid silica nanoparticles are typically hollow. However, it is not clear if, or how, amines like PEHA form stable liquid-like droplets in aqueous solution considering that they are small, highly soluble and nonamphiphilic molecules. We aim to investigate this issue. We are not aware of any prior study in this area. Ultimately, an understanding of aqueous polyamine clustering should help to inform future experimental studies on bioinspired nanosilica synthesis,^20^ on other systems involving amines (which are very common), and more generally on small-molecule solutions that display nanodroplets. We focus specifically on PEHA as a representative polyamine often used in bioinspired nanosilica production.

Classical molecular dynamics (MD) is a very powerful atomistic computational technique which, in principle, should be able to reproduce the clustering behavior of polyamines in dilute aqueous solutions. The use of MD simulations to study such clustering behavior is particularly important in the case of PEHA due to the fact that the best available technical-grade purity of PEHA is 90%, which makes experimental studies on its clustering behavior inconclusive without purification. Essentially, it is not currently known whether PEHA, or an accompanying impurity, forms the aqueous clusters observed in light-scattering experiments of dilute aqueous PEHA.^19^ Although it has been suggested that PEHA alone forms these clusters, the very soluble nature of PEHA and the likely presence of impurities casts significant doubt on this. Another difficulty is the range of protonation states available to PEHA in aqueous solution, and the pH range under which bioinspired nanosilica is observed to form. This is another issue MD simulation can potentially address, by observing whether clustering occurs for several different protonation states and for different types of counterion.

The remainder of this paper is structured as follows. First, we describe our computational methods, including our strategy to observe giant clustering and our selection of a suitable molecular force field. Since the employed force field is the main ingredient of any classical MD study, the choice of force field parameters is important and a nontrivial task in this case. Next, we describe our results for MD simulations of PEHA in several protonation states together with appropriate counterions and make comparison with relevant experimental data. Finally, we discuss the implications of our results for the synthesis of bioinspired nanosilica and more generally with respect to the understanding of equilibrium clustering of small molecules in solution.

## 2 Molecular Dynamics Simulation Details

We use large-scale classical MD simulations with explicit water to study the clustering behavior of PEHA in dilute aqueous solutions. We are interested in several experimentally relevant parameters including PEHA concentration and protonation state (which is related to the solution pH) and the counterion type. Therefore, we separately simulate samples with neutral, singly charged and doubly charged PEHA molecules, hereafter called PEHA, PEHA^+1^ and PEHA^+2^, respectively.

If pure PEHA is added to pure water, we expect the dominant protonation state and solution pH to be dependent on PEHA concentration; increasing PEHA concentration leads to higher pH. The counterion in this case is the hydroxide anion. Due to the high protonation equilibrium constants for primary amines, we expect for pH > 10 that neutral PEHA will dominate, while for near-neutral (pH 7) conditions we expect PEHA^+2^ to dominate. Clearly, PEHA^+1^ will be the dominant species for a range of pH between these limits. This covers the experimentally relevant pH range for bioinspired nanosilica production.^18,19^

The OH^–^ counterion can be partially exchanged by the addition of acid or salt. Addition of increasing concentrations of acid restores the pH balance toward neutral and shifts the dominant counterion toward the corresponding acid anion. Using salt instead mainly switches the counterion only; solution pH is less affected. Thus, solution pH (and therefore the dominant PEHA protonation state) and dominant counterion type can be adjusted relatively independently by the addition of varying amounts of acid or salt, assuming they feature the same anion. Here, for each simulated PEHA protonation state we perform simulations with two different counterions; chloride and acetate. Our simulations therefore correspond to the case where the hydroxide anion has been largely replaced as the dominant counterion by the addition of sufficient acid or salt, and to a wide range of solution pH. We also focus on dilute aqueous solutions, 200 and 400 mM, due to their experimental relevance.

It is not straightforward to determine whether, or why, large stable clusters form in a molecular simulation. Ideally, clusters will form spontaneously and quickly from a dispersion, evolving to form size-limited structures for which the free energy can be computed and compared to the dispersed state. However, in practice, spontaneous formation of large clusters can be frustrated by a large formation free energy barrier and occur only slowly, if at all. Moreover, exact free energy calculation methods have not been tested on simulated cluster fluid states, making the identification of the equilibrium structure problematic. A further problem caused by slow cluster dynamics is that it can also be difficult to distinguish whether a simulation containing a few large clusters corresponds to an equilibrium cluster state, or whether ripening of the cluster state toward bulk phase separation would occur in increasingly large simulations given sufficient time. To partially overcome these difficulties, in line with earlier work on the simulation of clustering in aqueous glycine,^9^ we therefore perform two simulations with different initial configurations for each simulated state.

One initial configuration corresponds to a dispersed state. But to overcome any potential cluster formation free energy barrier, we also perform simulations initiated from a preformed, large, single-cluster state. If the same final state is achieved in both simulations then it must be the equilibrium state, either dispersed or clustered, for that simulation size. If different final states are obtained for each simulation, we cannot decide which state is stable and which is metastable. Moreover, if the final state consists of a single large cluster, we cannot decide whether the same-size cluster or a larger cluster would occur in much larger simulations or whether bulk phase separation would occur in the thermodynamic limit.

**Figure 1:**
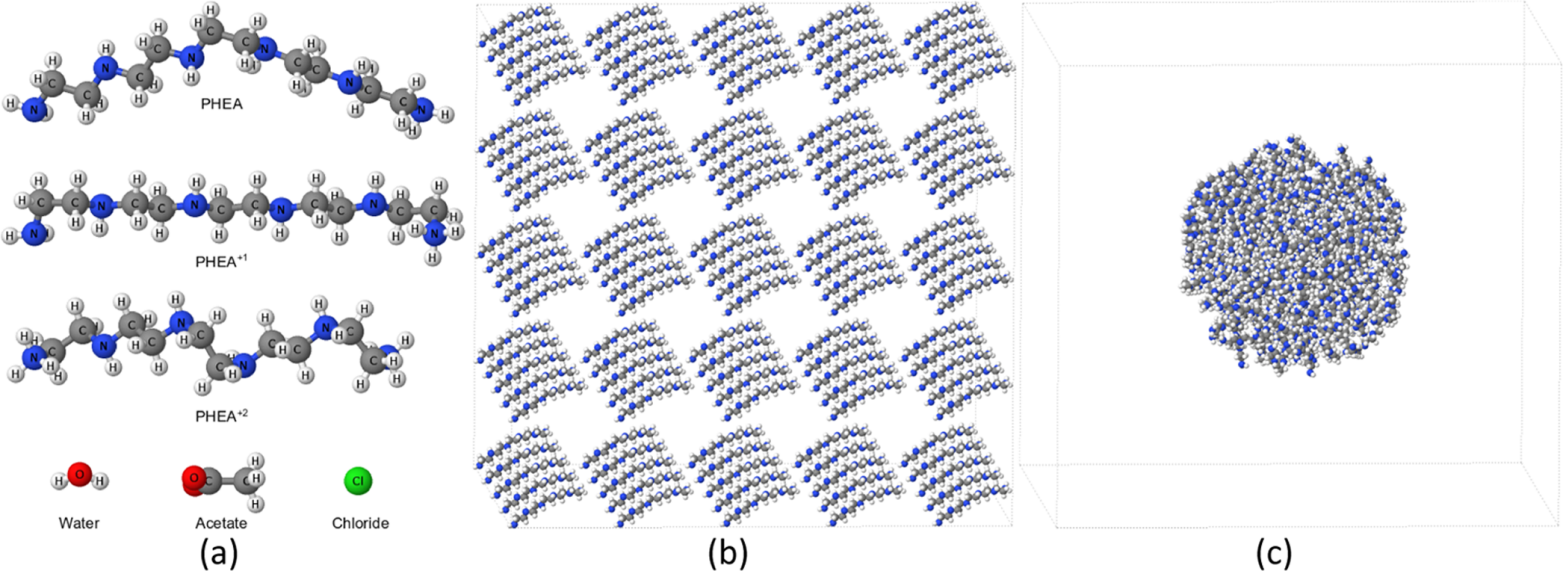
Initial geometries and typical initial states of PEHA in 200 and 400 mM aqueous solutions. (a) Optimized geometries of the different molecules involved in our molecular dynamics simulations. (b) Typical initial ordered configuration of a dispersed state. (c) Typical initial aggregated configuration of a clustered state. Cubic simulation boxes (with periodic boundaries) are shown here for the case of neutral PEHA molecules with all water molecules removed for clarity.

We therefore require two initial configurations corresponding to a dispersed state and a large single PEHA cluster for each PEHA protonation and concentration state and each counterion type. Typical dispersed and clustered initial states are shown in Figure 1b,c. Figure 1a reports the geometries of the different molecules involved in our molecular dynamics simulations. Singly charged PEHA is assumed to be protonated at one end, while, due to energetic considerations, doubly charged PEHA is protonated at both ends.

The search for an optimal set of force fields for water and PEHA is also not straightforward. However, when attempting to reproduce sensitive liquid-phase phenomena, such as clustering in the liquid phase, it is wise to use molecular models calibrated to reproduce other sensitive liquid-phase phenomena such as solubility constants and hydration free energies.

We therefore used the all-atom optimized potentials for liquid simulations (OPLS-AA) force field^21,22^ augmented with the 1.27*CM5 charge scheme for neutral PEHA,^23^ which requires the TIP3P model for water.^24,25^ Calibration of this charge scheme is based on rescaling atomic partial charges to reproduce experimental hydration free energies, heats of vaporization and densities for a wide range of neutral organic molecules. We apply it here directly to neutral PEHA, which was not part of the calibration set.

Considering that we also use charged species in our simulations, while the above calibration scheme is designed for neutral organic molecules, we adopted the following detailed strategy. For the chloride counterion, we used the OPLS-AA force field parameters from Yagasaki et al.^26^ and the partial atomic charge was set to −1. However, for consistency, partial atomic charges for the acetate counterion as well as PEHA^+1^ and PEHA^+2^ were recalculated based on the OPLS-AA force field parameters and the 1.27*CM5 charge scheme as follows. First, OPLS-AA molecular models for the acetate counterion and all PEHA molecular models were obtained from the LigParGen database.^27^ This database lists OPLS-AA force field parameters, partial atomic charges and molecular geometries, as desired, but unfortunately uses the 1.14*CM1A charge scheme for neutral molecules.^22^ As the 1.27*CM5 charge scheme has been shown to be slightly superior to the 1.14*CM1A scheme,^23^ we therefore scaled back the partial charges by 1.14 to revert back to the original OPLS-AA charges^21^ for neutral PEHA.

Before performing any first-principles calculations on the acetate counterion and the different PEHA variants, the geometry of each molecule was optimized using a separate classical molecular dynamics simulation. The purpose of this step is to ensure that the actual bond lengths and angles are located at their equilibrium values assumed by the OPLS-AA force field. In each simulation, one solute molecule was placed at the center of a simulation box containing 234 water molecules. During geometry optimization we used very large force constants and an extremely tight optimization stopping criteria to force bond angles and lengths to reach their equilibrium values. The above optimization step ensured that the final geometry of each molecule perfectly reflects the employed OPLS-AA force field parameters. The resulting optimized geometries were then used as input geometries for calculating the new CM5 charges in the GAUSSIAN code.^28^

In the following, we briefly describe our first-principles calculations to determine the 1.27*CM5 charges for the acetate counterion and the different PEHA molecules. To be able to use the 1.27 scaling factor of charges we followed the first-principles calculation protocol described by Marenich et al.^29^ and Vilseck et al.^30^ In our GAUSSIAN calculations, we employed the M06-2X^31^ hybrid density functional and the 6-311+G(2df,2p) Pople basis set^32^ in conjunction with the Hirshfeld population analysis method. It should be noted that our Gaussian calculations did not involve any further geometry optimizations. While the resulting CM5 partial atomic charges were used as they are in the case of the acetate counterion, PEHA+1, and PEHA+2 molecules, the CM5 charges for the neutral PEHA molecule were scaled by 1.27 in accordance with the 1.27*CM5 charge scheme. All results described below use these molecular force fields which incorporate our new partial charges consistent with the 1.27*CM5 charge scheme. Partial charges for each atom are given in Table 1.

**Table 1:** Atomic Partial Charges on the Different PEHA Variants, Acetate Counterion, and Water Molecule^a^

atom	PEHA	PEHA^+1^	PEHA^+2^	atom	acetate^–1^
N1	–0.876	–0.687	–0.575	C1(CO_2_)	0.144
H1(N1)	0.363	0.285	0.353	O1(CO_2_)	–0.554
H2(N1)	0.364	0.290	0.397	O1(CO_2_)	–0.560
H3(N1)			0.399	C(NH_3_)	–0.263
C1	–0.086	–0.069	–0.018	H1(NH_3_)	0.072
H1(C1)	0.114	0.085	0.134	H2(NH_3_)	0.081
H2(C1)	0.122	0.099	0.138	H3(NH_3_)	0.081
C2	–0.083	–0.068	–0.046	total	–1
H1(C2)	0.123	0.100	0.113		
H2(C2)	0.113	0.085	0.121	**atom**	**H_2_O**
N2	–0.679	–0.535	–0.501	O	–0.830
H(N2)	0.374	0.292	0.308	H	0.415
C3	–0.083	–0.065	–0.060	total	0
H1(C3)	0.130	0.103	0.106		
H2(C3)	0.108	0.085	0.102		
C4	–0.082	–0.066	–0.067		
H1(C4)	0.131	0.102	0.103		
H2(C4)	0.107	0.084	0.092		
N3	–0.659	–0.534	–0.543		
H(N3)	0.379	0.295	0.276		
C5	–0.079	–0.065	–0.066		
H1(C5)	0.127	0.103	0.093		
H2(C5)	0.110	0.084	0.104		
C6	– 0.084	–0.066	–0.065		
H1(C6)	0.123	0.103	0.088		
H2(C6)	0.115	0.084	0.104		
N4	–0.685	–0.532	–0.512		
H(N4)	0.350	0.296	0.299		
C7	–0.085	–0.062	–0.061		
H1(C7)	0.128	0.106	0.106		
H2(C7)	0.105	0.089	0.090		
C8	–0.085	–0.059	–0.058		
H1(C8)	0.129	0.108	0.108		
H2(C8)	0.105	0.092	0.095		
N5	–0.680	–0.499	–0.500		
H(N5)	0.374	0.311	0.311		
C9	–0.083	–0.043	–0.043		
H1(C9)	0.130	0.122	0.122		
H2(C9)	0.106	0.116	0.116		
C10	–0.086	–0.017	–0.017		
H1(C10)	0.106	0.138	0.139		
H2(C10)	0.129	0.135	0.136		
N6	–0.877	–0.574	–0.573		
H1(N6)	0.363	0.399	0.400		
H2(N6)	0.364	0.353	0.355		
H3(N6)		0.397	0.397		
total	0	–1	–2		

a Except for water, all charges correspond to the 1.27*CM5 charge scaling scheme.

Our classical MD simulations were carried out using the molecular dynamics simulation toolkit OpenMM^33^ version 7. We initially used the more popular large-scale atomic/molecular massively parallel Simulator (LAMMPS) code,^34^ but then switched to repeat all simulations using the OpenMM code. The use of OpenMM on a single graphic processing unit (GPU) allowed us to run very long simulations in a reasonable time. For example, it took LAMMPS about 3 days on 16 CPU cores to complete 30 ns, while it took OpenMM 1 day on 1 GPU to complete 100 ns of the same simulation. The results obtained from LAMMPS and OpenMM were almost identical, and thus we report only the OpenMM results.

Equations of motion were integrated using the Langevin Leapfrog integrator^35^ with a temperature of 298.15 K and a friction coefficient of 1 ps^–1^. Long-range Coulomb interactions were computed using the particle mesh Ewald (PME) method^36^ with a real space cutoff of 1.2 nm. The short-range interaction cutoff was also set to 1.2 nm. Since we did not use a flexible force field for water, all simulations employed a time step of 2.0 fs. Periodic boundary conditions (PBC) were applied in all directions to mimic bulk liquid samples. Simulation was carried out at a constant pressure of 1 bar, controlled by the Monte Carlo barostat,^37^ using a volume adjustment frequency of 25 time steps. After tight optimization of all initial geometries, a molecular dynamics simulation of each sample was run for 100.0 ns. Radial distribution function analysis was carried out using the MDAnalysis python package.^38^

Our cubic liquid simulation boxes contained 125 PEHA molecules and 29,250 water molecules in the case of 200 mM solutions, and 125 PEHA molecules and 14,625 water molecules in the case of 400 mM solutions. The number of added counterions was 125 and 250 for singly charged and doubly charged PEHA systems, respectively.

## 3 Simulation Results and Their Comparison with Experiments

**Figure 2:**
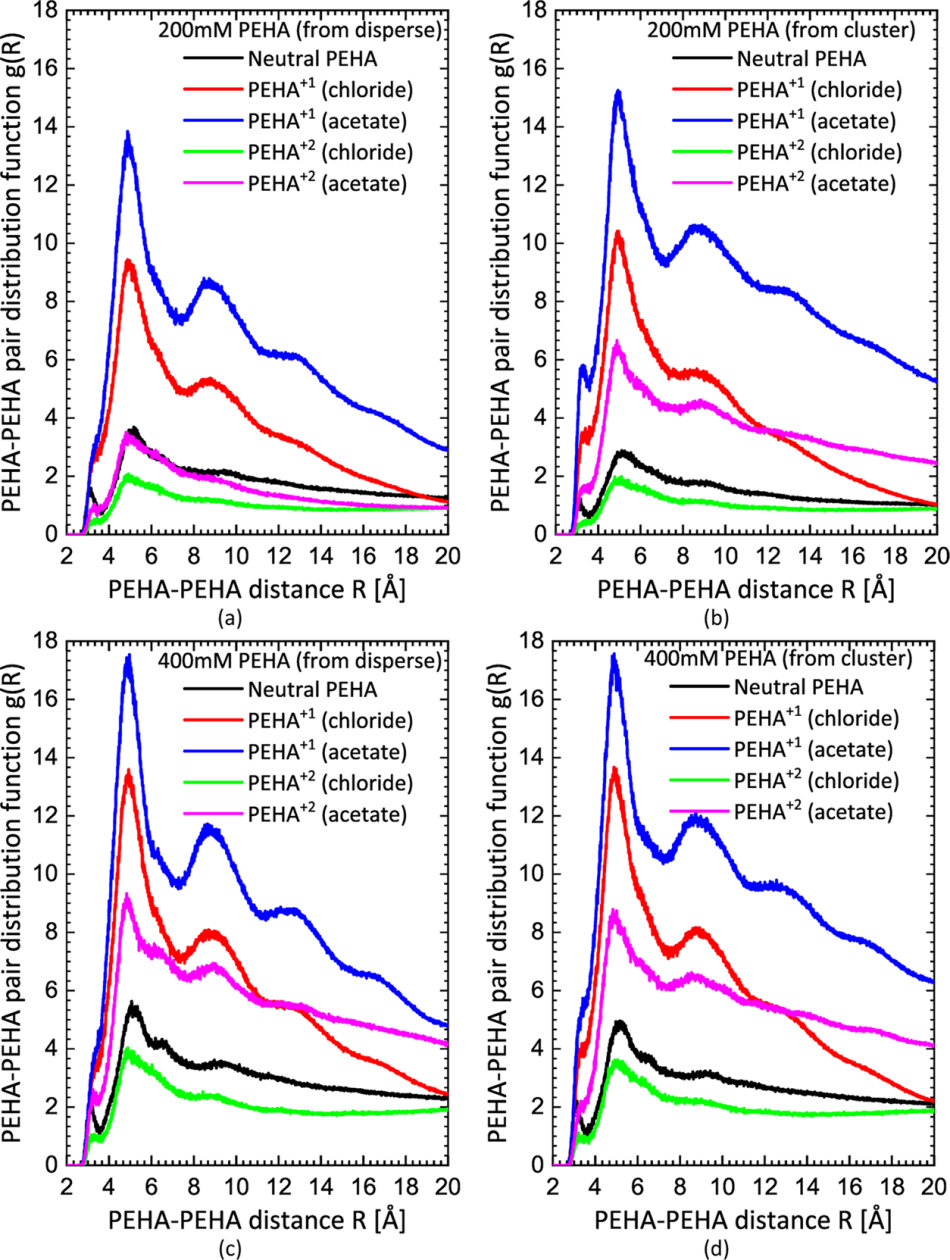
PEHA–PEHA pair distribution functions for neutral PEHA, PEHA^+1^, and PEHA^+2^ in 200 mM (a, b) and 400 mM (c, d) aqueous solutions initiated from dispersed (left) and preclustered (right) configurations.

**Figure 3:**
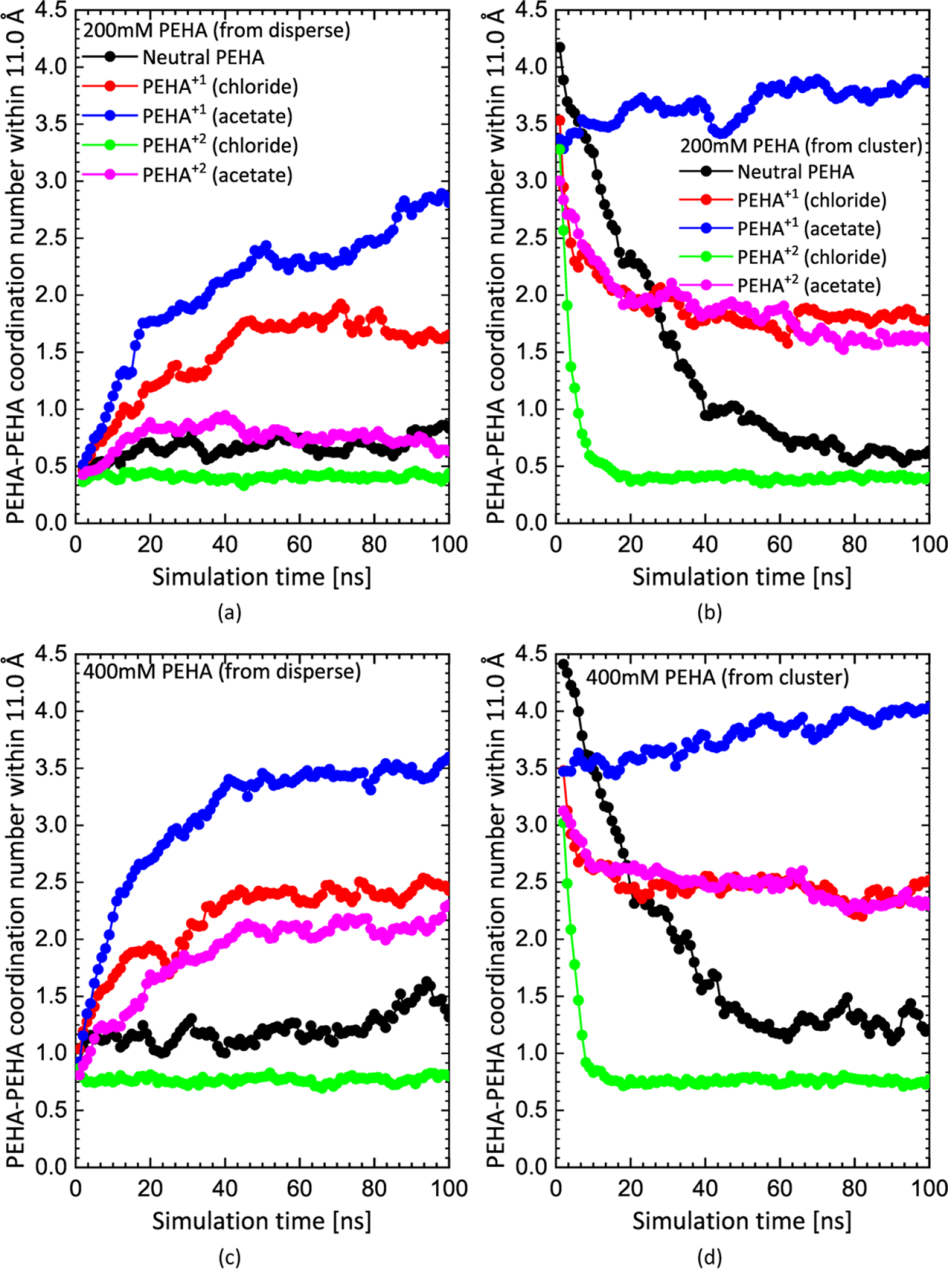
Evolution of the PEHA–PEHA coordination number (see text) for neutral PEHA, PEHA^+1^, and PEHA^+2^ in 200 mM (top) and 400 mM (lower) aqueous solutions initiated from dispersed (left) and clustered (right) configurations.

**Figure 4:**
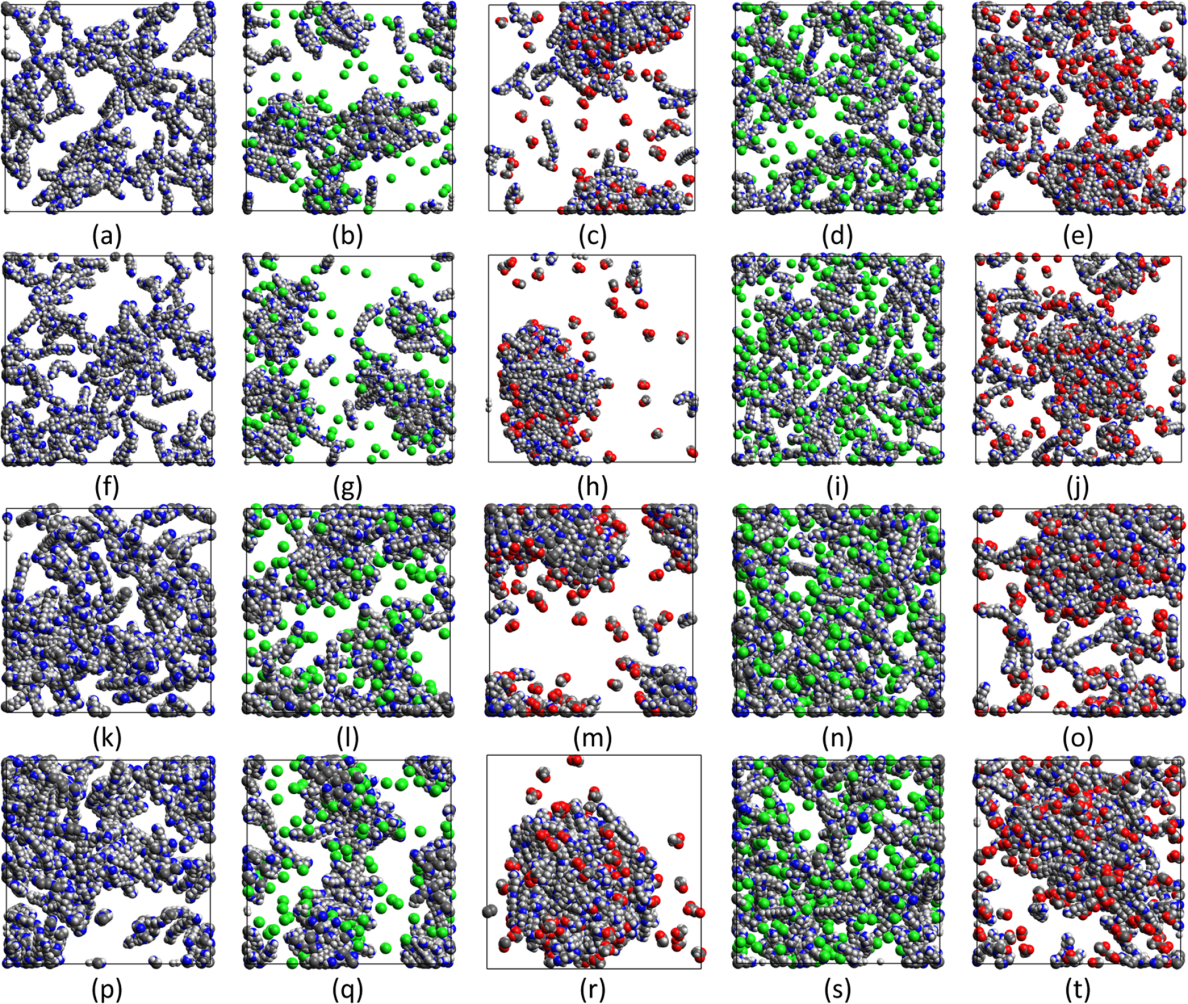
Final configurations (i.e., after 100 ns) for the simulations corresponding to Figure 2 with water removed to aid the comparison. The five columns, from left to right, correspond to neutral PEHA, PEHA^+1^ + chloride, PEHA^+1^ + acetate, PEHA^+2^ + chloride and PEHA^+2^ + acetate. The four rows, top-to-bottom, correspond to 200 mM initiated from a dispersion, 200 mM initiated from a single large cluster, 400 mM initiated from a dispersion and 400 mM initiated from a single large cluster.

We performed simulations of PEHA at two concentrations (200 and 400 mM) in three different charge states (neutral PEHA, PEHA^+1^, and PEHA^+2^), for two different counterions (chloride and acetate), starting from two different initial states (dispersed and clustered). In Figure 2, we report the final PEHA–PEHA radial distribution functions *g*(*R*) obtained for the 200 and 400 mM concentrations when they were initiated from dispersed and clustered configurations. In Figure 3, we report the time evolution of the PEHA–PEHA coordination number for each simulation calculated by integrating the radial distribution function, *g*(*R*), up to a distance of 1.1 nm. This pair distribution function is the average of the partial pair distribution functions between each of the six nitrogen atoms in each PEHA molecule and their counterparts in all other PEHA molecules. The cutoff of 1.1 nm is chosen for the coordination number because this includes the main peak and second peak in *g*(*R*) (see Figure 2). Therefore, the coordination number effectively measures the average number of nearest and next-nearest neighbors around each PEHA molecule and is a useful measure of clustering. When clusters are absent, the coordination will normally be <1. Figure 4 shows snapshots of the corresponding final molecular dynamics configurations, where all water molecules have been removed and box dimensions are equalized for clarity.

Starting with solutions of neutral PEHA molecules, we see that neutral PEHA appears to form a nearly uniform dispersion at 200 mM. According to the evolution of the coordination number in Figure 3b, corresponding to simulations started with a single large cluster, the neutral PEHA cluster completely dissolves before 100 ns. The snapshot in Figure 4f confirms this. Similar results are obtained for the 400 mM system (see Figures 3d and 4p). Although the coordination number for the 400 mM system converges to just above 1 in Figure 3d, this indicates the existence of numerous pairs or short chains of neutral PEHA at this higher concentration and not large clusters. Indeed, the equilibrium coordination number for neutral PEHA at 400 mM is about twice that at 200 mM, which is entirely expected for a dispersed system. Therefore, neutral PEHA does not appear to form large clusters, even at 400 mM.

These simulations with neutral PEHA correspond approximately to the experimental case of high pH > 10 where the neutral PEHA form dominates. Actually, there will still exist in experiments at high pH a low concentration of charged PEHA^+1^ ions balanced by hydroxide ions (OH^–^), which are not included in these simulations. Nevertheless, any remaining OH^–^ ions will be very soluble and unlikely to influence clustering. Meanwhile, any remaining PEHA^+1^ ions, since they exhibit mutual coulomb repulsions, will likely increase the tendency for PEHA to disperse through their cross-interaction with neutral PEHA. Therefore, these simulations of neutral PEHA probably underestimate the tendency for PEHA to disperse in aqueous solutions at high pH. Experimentally, results are not recorded for clustering of aqueous PEHA at such high values of pH,^10^ so these simulation results are a prediction. However, the experimental results do show cluster size increasing between pH 9 and 7, from around 200 nm at pH 9 to over 1 μm at pH 7. If neutral PEHA alone was responsible for the formation of these giant clusters, we would expect to see the opposite trend since the concentration of neutral PEHA reduces as pH increases. Therefore, these simulation results agree with the observed experimental trend of rapidly reducing cluster size with increasing pH.

Regarding singly charged PEHA molecules (PEHA^+1^), we clearly see that they form clustered states at 200 mM with the chloride counterion. The coordination numbers for this system at 200 mM in Figure 3a,b, corresponding to dispersed and clustered initial states, respectively, bracket the equilibrium state, which judging from the final snapshots in Figure 4b,g, consists of relatively small, isolated clusters 2–5 nm in diameter (the end-to-end length of PEHA is ∼1.5 nm). At 400 mM, the coordination number plots in Figure 3c,d indicate that slightly larger clusters are formed at equilibrium than at 200 mM. The final snapshots in Figure 4l,q (initiated from dispersed and clustered states, respectively) tend to confirm this. An interesting observation in all of these simulations is that chloride appears mainly to coat the surface of these PEHA^+1^ clusters, or remain dispersed, rather than penetrating them.

However, clustering is much enhanced for PEHA^+1^ with the less soluble acetate counterion. This system shows the strongest tendency for cluster formation among all of the simulations. The coordination number for both the 200 and 400 mM simulations starting with a single large PEHA^+1^ cluster plus acetate increases gradually in Figure 3b,d, respectively. These large clusters are very obvious in the snapshots (Figure 4h,r). Both these simulations suggest either a larger cluster is stable or bulk phase separation might occur in the thermodynamic limit. In experiments, bulk phase separation would be observed as precipitation. As this is not actually observed, it suggests the clusters seen in the simulations might have a finite upper size if much larger simulations could be performed.

Experimentally, these simulations correspond to intermediate pH, ca. 8–9, with a combination of acid and salt added at nearly the same concentration as PEHA. The hydroxide ion concentration at this pH is not significant relative to the PEHA concentration. At this pH, experiments with aqueous PEHA show that giant clusters are apparently stable with cluster diameter <1 μm. It is possible, therefore, that these experimentally observed clusters correspond to clusters of PEHA^+1^ together with a relatively insoluble salt anion.

However, for doubly charged PEHA molecules (PEHA^+2^), we see that the higher charge state tends to disrupt the formation of clusters due to coulombic repulsion. A dispersed state is clearly stable at 200 mM with the chloride counterion since the large single cluster quickly dissolves in Figure 3b. By the end of the simulation, Figure 4i indicates a relatively homogeneous dispersion. From Figures 3d and 4s, we see a similar dispersed state is attained by the end of the simulation at the higher 400 mM concentration with the chloride counterion.

On the other hand, when the less soluble acetate counterion is used, the coordination numbers in Figure 3a,b bracket the equilibrium state. Therefore, it cannot be decided from these simulations alone whether large PEHA^+2^ + acetate clusters are stable at 200 mM. At 400 mM, however, with the acetate counterion the situation is somewhat clearer as the coordination numbers in Figure 3c,d indicate that acetate can stabilize PEHA^+2^ clusters at this concentration. Likewise, large clusters are observed in the final snapshots in Figure 4o,t.

These simulations with PEHA^+2^ correspond to experiments at near-neutral conditions where a combination of twice as much acid and salt, relative to PEHA, has been added. Large clusters several microns in diameter are observed in experiments in this case.^10^ Again, the simulations suggest that these clusters might be explained by PEHA^+2^ forming size-limited clusters with a relatively insoluble acid/salt anion.

## 4 Discussion and Conclusions

Overall, these simulations suggest that PEHA can form large stable clusters in aqueous solution with added acid or salt over a suitable range of pH. Importantly, the clusters are not formed by PEHA alone; neutral PEHA readily disperses and counterions are needed to bind together charged PEHA. We also find that counterion type is important, with clustering enhanced through the use of less soluble acid/salt anions compared to chloride.

However, these simulations do not prove that the experimentally observed clusters, which are orders of magnitude larger than those observed in our simulations, are formed mainly of PEHA as our results might be sensitive to the force fields used, although we have tried to mitigate against this possibility using the best calibrated force fields available for PEHA.

Moreover, considering the high proportion of impurities in PEHA as supplied, we cannot rule out the possibility that the experimentally observed clusters are caused primarily by these impurities. Nevertheless, the hypothesis that the clusters observed in experiments are caused primarily by PEHA binding with relatively insoluble counterions is reasonable, worth exploring further, and a sensible basis for understanding the manufacture of bioinspired nanosilica until it is shown to be wrong. Clearly, future experiments conducted with purified reagents would be very beneficial in this debate.

Together with our earlier work on aqueous glycine,^9^ this work shows how the general problem of understanding giant clustering, i.e., the formation of nanodroplets, in nonamphiphilic small-molecule solutions can be tackled. Our approach uses MD simulations that employ the latest GPU-enhanced codes together with molecular force fields optimized for aqueous phase properties. This enables us to simulate to convergence in a reasonable time the evolution of large solute clusters in relatively large aqueous systems. Another key aspect of this work is the use of two different initial states, dispersed and clustered, to help overcome any high cluster nucleation barriers and therefore determine which is the equilibrium state. The use of fast GPU-based MD codes makes such simulations much more accessible. Our previous work on glycine used instead a CPU-based MD code which limited the opportunity to reach convergence in those simulations.

It is worth understanding how large size-limited clusters can form in our simulations of aqueous PEHA. Our simulations show that, by itself, neutral PEHA does not appear to exhibit sufficiently strong attractive effective interactions in water to aggregate. Even up to 400 mM, neutral PEHA is apparently quite soluble. Therefore, due to Coulombic repulsion, the charged versions of PEHA will also not aggregate by themselves. However, our simulations suggest that the addition of counterions can lead to large clusters of PEHA^+1^ and PEHA^+2^ under suitable conditions. In other words, coulomb attraction between charged PEHA and counterions can, it seems, provide an effective short-range attraction, sufficient to overcome mutual coulomb repulsion at short-range and thereby cause aggregation between charged PEHA molecules. However, for clusters to be stable and size-limited, PEHA and the counterion must exhibit unequal solubilities within the cluster, i.e., one of these species must favorably partition within the cluster such that long-range coulomb repulsion between charged PEHA molecules limits cluster growth beyond a specific cluster size. This appears to be realized in our simulations since the counterions tend to decorate the surfaces of the clusters rather than penetrating them.

It is well known that the SALR (short-range attraction, long-range repulsion) mechanism can generate large, stable, size-limited clusters.^5,39^ In our case, we suggest the counterions generate an effective short-range attraction between charged PEHA molecules such that effective PEHA–PEHA interactions might be modeled as SALR interactions. Therefore, it should be possible by altering the counterion type and concentration, through adjusting the type and concentration of added acid and/or salt in experiments, to tailor the cluster size and concentration. This mechanism might be modeled effectively in terms of the SALR cluster fluid.

Taking a wider perspective, we view the clusters formed in our simulations as kinds of complex coacervate. Such coacervates are known to form in solutions of oppositely charged long polyion mixtures, such as polyethylenimine (PEI)–DNA mixtures, where they appear to form macroscopic liquid-like droplets dispersed in solution.^6^ Their properties are known to be sensitive to a wide range of parameters. But there is no reason in principle why such complexes cannot form through the aggregation of much smaller solutes, like PEHA, as a result of the same physical principles, leading to the formation of correspondingly smaller, stable, size-limited liquid-like droplets.

Alternatively, the aggregation process in our simulations can also be viewed as a limited kind of “salting-out” or electrostatic flocculation process. However, instead of generating macroscopic solid particles that eventually settle under gravity, only microscopic liquid-like droplets are formed. Given their size and similar mass density to the solvent, they are readily dispersed and remain suspended in solution.
